# Political commitments needed to address health impacts of the climate crisis and biodiversity loss

**DOI:** 10.2471/BLT.22.289591

**Published:** 2023-02-01

**Authors:** Viroj Tangcharoensathien, Diarmid Campbell-Lendrum, Peter Friberg, Angkana Lekagul

**Affiliations:** a International Health Policy Program, Ministry of Public Health, Tiwanond Road, Muang District, Nonthaburi 11000, Thailand.; b Department of Environment, Climate Change and Health, World Health Organization, Geneva, Switzerland.; c Swedish Institute for Global Health Transformation, Stockholm, Sweden.

Climate change causes metrological events and extreme weather that are associated with the increase of multiple adverse health outcomes. The most common such health outcomes are infectious vector-borne, foodborne and waterborne diseases, and respiratory, cardiovascular or neurological diseases.^1^ Climate change also exposes humans to emerging infectious diseases; for example, melting Arctic ice may release viruses and bacteria trapped in glaciers, and changes to animal habitats and migration may introduce new disease vectors.^2^

Despite increasing global attention to the health impacts of climate change, international commitments and action remain inadequate. Progress reported to the United Nations Framework Convention on Climate Change Conference of Parties (COP) 27 was hampered by the European energy crisis as some European countries turned to fossil energy, although 26 countries committed to curb greenhouse gas emissions by 43% by 2030.^3^

The COP15 to the Convention on Biological Diversity boosted international attention to global biodiversity loss. For example, the world’s largest inland fishery, the Mekong River Basin, is undergoing massive hydropower development. When the construction of 78 dams in tributaries and the main Mekong River is completed, biodiversity loss and decreased fish biomass will adversely affect vulnerable population groups whose livelihoods depend on natural fishing in this river system.^4^^,^^5^ The construction of these dams will lead to major social and environmental impacts, including population relocation and loss of agricultural land and cultural life.^6^ Despite the major transboundary impacts of dam construction, collective solutions across countries are still inadequate. The non-legally binding Mekong Agreement mandates international consultations before constructing main-stem dams, but tributary dams fall within national jurisdictions. Therefore, only a notification to the Mekong River Commission is needed for construction to go ahead.

Despite the continued impacts on biodiversity, progress in implementing the global biodiversity framework has been hampered by national-level inaction. Parties to the biodiversity summit in December 2022 agreed on two major targets: protect and restore 30% of the world’s land and seas globally by 2030 while respecting the rights of indigenous peoples who depend on and steward much of Earth’s remaining biodiversity, and reduce the extinction rate of animal species by 10-fold for all species by 2050.^7^

Although air pollution has decreased significantly in Europe over the last three decades,^8^ none of the world’s 195 countries and territories met the World Health Organization’s (WHO) guideline for air quality of 10 μg/m^3^/annum between 1990 and 2017.^9^ Despite the detrimental effects of air pollution on human life, evidence on effective interventions for improved air quality on health is inconclusive – due to the heterogeneity of interventions, methods and outcome measurements.^10^ Nevertheless, the potential health gains of addressing air pollution are immense. As shown in this issue of the *Bulletin of the World Health Organization*, if Member States in the WHO Western Pacific Region were able to achieve the more stringent WHO particulate matter (PM)_2.5_ air quality targets of < 5 µg/m^3^/annum, 3.1 million annual deaths would be averted.^11^ WHO urges all Member States to act, including by making air quality data publicly available, setting legally binding standards and goals for air quality management at international, national and local levels, and harmonizing air quality standards and air quality management systems, guided by local epidemiology and sources of PM_2.5_.^12^

Digital technologies, such as cloud-based data storages, streaming content over the internet and mining of bitcoin, can aggravate climate change if they use fossil fuels to generate electricity. For example, more than half of the global energy used by bitcoin mining is from fossil fuels. Each 1 United States dollar (US$) of bitcoin market value created through mining was associated with US$ 0.49 in combined health and climate damages in the United States.^13^ Political commitments are needed to ensure clean energy is provided for the increasing use of modern technologies.

The climate crisis will pose a heavy burden and generate long-term impacts on the world’s next generation. The global movement of youth-led climate intervention has gained momentum over the past few years;^14^ however, governments and partners need to support this movement to strengthen capacity to take action, enlist broader constituent groups, and form networks to safeguard nature.

This theme issue of the *Bulletin *was launched at the Prince Mahidol Award Conference 2023. Evidence contributes to informing policy and practice to mitigate the impacts of the climate crisis on biodiversity and human health. However, global collaboration and sustained political leadership, supported by legally binding agreements, incentives, regulation and monitoring, as well as the involvement of youth are essential.
